# Morphological Transformation of Peptide Nanoassemblies through Conformational Transition of Core-forming Peptides

**DOI:** 10.3390/polym11010039

**Published:** 2018-12-28

**Authors:** Tomonori Waku, Naoyuki Hirata, Masamichi Nozaki, Kanta Nogami, Shigeru Kunugi, Naoki Tanaka

**Affiliations:** Faculty of Molecular Chemistry and Engineering, Kyoto Institute of Technology, Gosyokaido-cho, Matsugasaki, Sakyo-ku, Kyoto 606-8585, Japan; h.naoyuki332@gmail.com (N.H.); m.nozaki.kit@gmail.com (M.N.); kotetuzeroshiki@i.softbank.jp (K.N.); kunugi@snr.kit.ac.jp (S.K.); tanaka@kit.ac.jp (N.T.)

**Keywords:** aromatic peptides, self-assembly, morphological change, secondary structure

## Abstract

Morphological control of nanostructures that are composed of amphiphilic di- or tri-block molecules by external stimuli broadens their applications for molecular containers, nanoreactors, and controlled release materials. In this study, triblock amphiphiles comprising oligo(ethylene glycol), oligo(l-lysine), and tetra(l-phenylalanine) were prepared for the construction of nanostructures that can transform accompanying α-to-β transition of core-forming peptides. Circular dichroic (CD) measurements showed that the triblock amphiphiles adopted different secondary structures depending on the solvent environment: they adopt β-sheet structures in aqueous solution, while α-helix structures in 25% 2,2,2-trifluoroethanol (TFE) solution under basic pH conditions. Transmission electron microscopic (TEM) observation revealed that the triblock amphiphiles formed vesicle structures in 25% TFE aq. Solvent exchange from 25% TFE to water induced morphological transformation from vesicles to arc-shaped nanostructures accompanying α-β conformational transition. The transformable nanostructures may be useful as novel smart nanomaterials for molecular containers and micro reactors.

## 1. Introduction

Amphiphilic di- or tri- block type molecules with hydrophilic and hydrophobic segments can self-assemble into various core-shell type nanostructures, such as micelles, vesicles, tubes, and fibers [1]. Morphological control of these nanostructures by external stimuli broadens their applications for molecular containers, nanoreactors, and controlled release materials. Therefore, various molecular designs have been reported for the construction of such smart nanomaterials that can respond to changes in the external environment, including pH, temperature, and solvent composition [2,3,4]. Among these, amphiphilic molecules, consisting of polypeptides, have been widely used as a building block for smart nanomaterials, as they exhibit a transition of secondary structures: α-helix, β-sheet, and random conformations [5,6,7,8]. The secondary structure transition of core-forming polypeptides can induce further drastic morphological changes, in contrast to that of shell-forming ones, which usually leads to changes only in the diameter of the nanostructures [9,10,11,12,13]. To date, many studies on the morphological changes based on the conformational transition of core-forming peptides have been reported. However, most studies have focused on the switching between assembly and disassembly, triggered by the structural α-to-random [14] or β-to-random transitions [15], which accompanies a sharp change in solubility. On the other hand, significantly less attention has been paid to the morphological change that is driven by α-to-β transition of core-forming hydrophobic polypeptides [2], although such conformational transition can be expected to induce a drastic morphological transition from one morphology to another morphology. Definite conformation of the building blocks both before and after the transformation allows us to precisely design their nanoassemblies. Therefore, the nanostructures that can transform accompanying α-to-β transition of core-forming peptides can open new opportunities for the applications of smart nanomaterials.

In nature, some proteins often change their aggregation state, which is accompanied by their conformation transition. For example, the process of amyloid fibril formation would be involved in α-β conformational transformation of the native proteins, resulting in a high content of beta-sheet structures [16]. Some amyloidogenic proteins have specific core sequences of several amino acid residues within the protein, which often contain aromatic amino acids and are originally prone to adopt β-sheet conformation [17,18]. It is believed that the whole α-β conformational transition of amyloidogenic proteins is triggered by the local transition of the core region [19,20]. Mimicking this characteristic, we designed triblock conjugates as model molecules, which are composed of oligo(ethylene glycol), oligo(l-lysine), and tetra(l-phenylalanine), for the construction of the smart nanomaterials possessing transformation ability thorough α-β conformational transition. Phenylalanine-based aromatic peptides have been widely used as a self-assembling motif for the construction of β-sheet assemblies, since the aromatic stacking interactions may play an important role for molecular assembly that leads to the formation of amyloid-like nanofibers [21,22,23,24,25]. Therefore, the introduction of tetra(l-phenylalanine) would affect the secondary structure of the peptide segments. Oligo(l-lysine) normally exhibits a random-helix transition in response to the pH environment at room temperature [26].

Herein, we report a morphological transformation of the core-shell nanostructures composed of the triblock PEG-peptide amphiphiles thorough secondary structure transition of core-forming peptides. The triblock PEG-peptide amphiphiles exhibited different secondary structures that are dependent on the solvent-environment. When peptide segments adopt α-helix, the triblock conjugates formed vesicle structures. In addition, the α-β transition of the core-forming peptides induced by changing the solvent-environment resulted in morphological transition from vesicles to arc-shaped nanostructures.

## 2. Materials and Methods 

### 2.1. Materials

39-amino-*N*-(9-fluorenylmethoxycarbonyl)-4,7,10,13,16,19,22,25,28,31,34,37-dodecaoxanonatriacontanoic acid (Fmoc-*N*-amido-dPEG_12_ acid) was purchased from Quanta BioDesign Ltd. (Plain City, OH, USA). 2-chlorotrityl resin, *N*,*N*-diisopropylethylamine (DIPEA), *N*-α-(9-Fluorenylmethoxycarbonyl)-*N*-ε-(*tert*-butoxycarbonyl)-l-lysine (Fmoc-Lys(Boc)-OH), *N*-α-(9-Fluorenylmethoxycarbonyl)-l-phenylalanine (Fmoc-Phe-OH), 1-[bis(dimethylamino)methylene]-1*H*-benzotriazolium 3-oxide hexafluorophosphate (HBTU), 1-hydroxybenzotriazole (HOBT), and piperidine were purchased from Watanabe Chemical Industries Ltd. (Hiroshima, Japan). *N*,*N*-dimethylformamide (DMF), isopropanol, methanol, diethyl ether, hexafluoroisopropanol (HFIP), dichloromethane (CH_2_Cl_2_), and trifluoroacetic acid (TFA) were purchased from Wako Pure Chemical Industries, Ltd. (Osaka, Japan). 2,2,2-trifluoroethanol (TFE) was purchased from Nacalai Tesque Inc. (Kyoto, Japan).

### 2.2. Synthesis of PEG-Peptide Amphiphiles

**Loading of resin**: Fmoc-*N*-amido-dPEG_12_ was dehydrated by an azeotrope with benzene prior to use. A solution of Fmoc-*N*-amido-dPEG_12_ acid (0.238 mmol) and DIPEA (0.952 mmol) in CH_2_Cl_2_ (2.6 mL) was reacted with 2-chlorotrityl resin (0.397 mmol, 1.5 mmol/g loading max) for 12 h.

**Peptide synthesis**: Coupling reactions were conducted using standard Fmoc protocol. The coupling cycle included Fmoc deprotection (20% piperidine in DMF, 5 min, repeated three times), DMF wash, amino acid coupling: l-Fmoc amino acids (4 eq.), HBTU (3.6 eq.), HOBt (4 eq.), and DIPEA (8 eq.) for 30 min repeated twice, and DMF washed. After all coupling reactions, the obtained peptides were cleaved from the resin by H_2_O/TFA (5: 95 in volume) for 2 h. Subsequently, the peptide was precipitated in ice cold diethyl ether and filtered, centrifuged, and washed with diethyl ether. The crude peptides were purified by reversed-phase high-performance liquid (RP-HPLC). MS (MALDI-TOF): K_20_-EG_12_; Cald. MASS: 3182.05, Obsd. MASS: 3182.28, K_16_F_4_-EG_12_; Cald. MASS: 3257.90, Obsd. MASS: 3256.10, F_4_K_16_-EG_12_; Cald. MASS: 3257.90, Obsd. MASS: 3256.87.

### 2.3. Self-Assembly of PEG-Peptide Amphiphiles in 25 vol % TFE Aqueous Solution

To obtain a homogeneous solution of monomeric peptide, the following procedure was used: F_4_K_16_-EG_12_ was dissolved in TFA, sonicated for 5 min, and then dried with nitrogen flow; then, the obtained film was re-dissolved in HFIP, sonicated for 10 min, and dried with nitrogen flow. The HFIP-treated film of F_4_K_16_-EG_12_ was dissolved in TFE at a concentration of 3.7 mM. The 750 µL NaHCO_3_ buffer (33 mM, pH 10.7) was added dropwise into a 250 µL of TFE solution containing F_4_K_16_-EG_12_ with gentle stirring at the rate of 20 µL/5 min. The obtained aggregates were analyzed with transmission electron microscopic (TEM), circular dichroic (CD), and dynamic light scattering (DLS). The self-assembly of the other PEG-peptide amphiphiles (K_20_-EG_12_, K_16_F_4_-EG_12_) was also investigated in a similar manner. 

### 2.4. Morphological Transition of Vesicles Consisting of PEG-Peptide Amphiphiles by Solvent Exchange

The dispersion of F_4_K_16_-EG_12_ vesicle in 25% TFE aqueous solution was dialyzed against NaHCO_3_ at various pH values to remove TFE. The resulting nanostructures were observed with TEM and their secondary structures were characterized with CD spectroscopy.

### 2.5. Measurements

#### 2.5.1. CD Measurements

CD measurements were performed on a J-720 spectropolarimeter (JASCO Applied Sciences, Halifax, NS, Canada), with an optical cell of 0.1 cm optical path length at 25 °C. The HFIP-treated film of the PEG-peptide amphiphiles was dissolved in water or in 25 vol % TFE aqueous solution at the concentration of 77 µM. The pH of the solution was adjusted with NaOH aq. prior to measurement. The helical content was calculated while using the following equation [27].

Helical content (%) = ([*θ*_208, obs_] − [*θ*_208, 0_]/[*θ*_208, 100_] − [*θ*_208, 0_]) × 100

where [*θ*_208, 0_] is −4600 deg⋅cm^2^·dmol^−1^ and [*θ*_208, 100_] is −33,000 deg⋅cm^2^·dmol^−1^.

#### 2.5.2. DLS Analysis

DLS analysis was performed using a DLS 7000 (Otsuka Electronics Co., Ltd., Osaka, Japan) at 25 °C. The light source was a He-Ne laser (630 nm) set at an angle of 45°. Experimental data were analyzed using the NNLS algorithm that was provided by the manufacturer.

#### 2.5.3. TEM Observation

The samples were negatively stained with 0.1% phosphotungstate, which was adjusted to pH 7.0 using sodium hydroxide. TEM measurements were performed using a JEM-1200EX II (JEOL, Tokyo, Japan), with an acceleration voltage of 85 keV.

#### 2.5.4. Atomic Force Microscopic (AFM) Observation

The topologies of the nanostructures consisting of F_4_K_16_-EG_12_ were visualized with a Nanoscope IIIa (Veeco Instruments, Santa Barbara, CA, USA) in tapping mode, and the cantilever was set vibrating in the z-direction at a resonance frequency of 290 kHz. The images were captured in air under ambient conditions using silicon tips.

## 3. Results and Discussion

**Figure 1:**
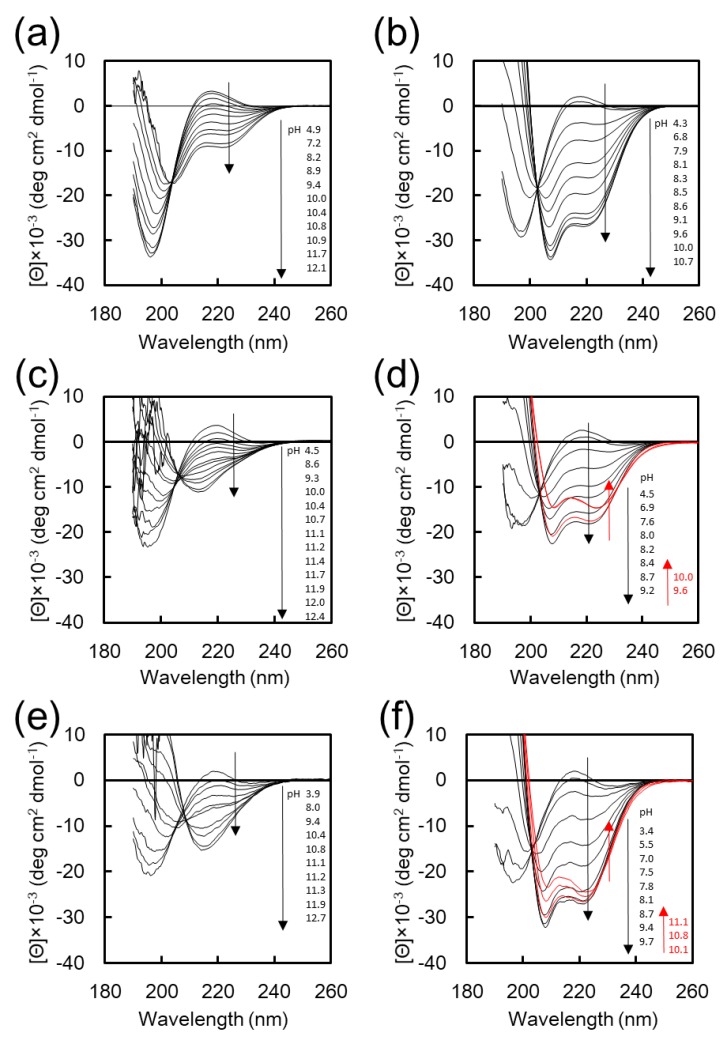
Circular dichroic (CD) spectra of K_20_-EG_12_ (**a**,**b**), K_16_F_4_-EG_12_ (**c**,**d**), and F_4_K_16_-EG_12_ (**e**,**f**). The measurements were performed in water (**a**,**c**,**e**) and in 25% 2,2,2-trifluoroethanol (TFE) aqueous solution (**b**,**d**,**f**) at various pHs at room temperature.

Three types of PEG-peptide amphiphiles, K_20_-EG_12_, K_16_F_4_-EG_12_, and F_4_K_16_-EG_12_, were synthesized by Fmoc solid phase synthesis, and their secondary structures and self-assembly behaviors were investigated (Table 1). Figure 1 shows the CD spectra of K_20_-EG_12_, K_16_F_4_-EG_12_, and F_4_K_16_-EG_12_ at various pH values in water and in 25% TFE aq. TFE is a well-known helix-assisted solvent [28]. CD spectra of K_20_-EG_12_ in water and in 25 vol % TFE aq. show that the intensity of negative cotton peaks at 222 and 208 nm, which are characteristic of α-helix, was increased with increasing pH through an isodichroic point at around 203 nm (Figure 1a,b) [27]. These results indicate that K_20_-EG_12_ undergoes a structural transition from random to α-helix with increasing pH, both in water and in 25% *v*/*v* TFE aq., although the helix content is low in water (Figure S1). In contrast, the transition behavior of K_16_F_4_-EG_12_ and F_4_K_16_-EG_12_ with increasing pH was quite different depending on the solvent. In the CD spectra of K_16_F_4_-EG_12_ and F_4_K_16_-EG_12_ in water, the intensity of the negative cotton peaks at 217 nm, characteristic of the β-sheet [27], was increased with increasing pH, indicating the transition from random to β-sheet conformation (Figure 1c,e, Figure S2). On the other hand, in 25% *v*/*v* TFE, the intensity of negative cotton peaks at 222 and 208 nm was increased, indicating the pH-responsive transition from random to α-helix conformation (Figure 1d,f). These results clearly indicate that the F_4_ segment significantly affects the secondary structure of K_16_F_4_-EG_12_ and F_4_K_16_-EG_12_ in basic aqueous solution. The pH dependences of the helicity for these peptide conjugates in 25% TFE are shown in Figure S1a. The helicity for all peptides has a maximum value and slightly decreases in the range of higher basic pH, which is possibly due to the low solubility of these peptides under the basic pH condition. More particularly, the maximum helicity for K_16_F_4_-EG_12_ in 25% TFE was much smaller than that for F_4_K_16_-EG_12_ and K_20_-EG_12_, indicating that K_16_F_4_-EG_12_ formed some aggregates. These results show that F_4_K_16_-EG_12_ adopts different secondary structures depending on the solvent environment without forming large aggregates; these adopt β-sheet structures in aqueous solution and α-helix structures in 25% TFE solution under basic pH condition. Thus, F_4_K_16_-EG_12_ is well suited as a component for the construction of smart nanoassemblies whose transformation is accompanied by a secondary structure transition in response to the solvent environment. 

**Figure 2:**
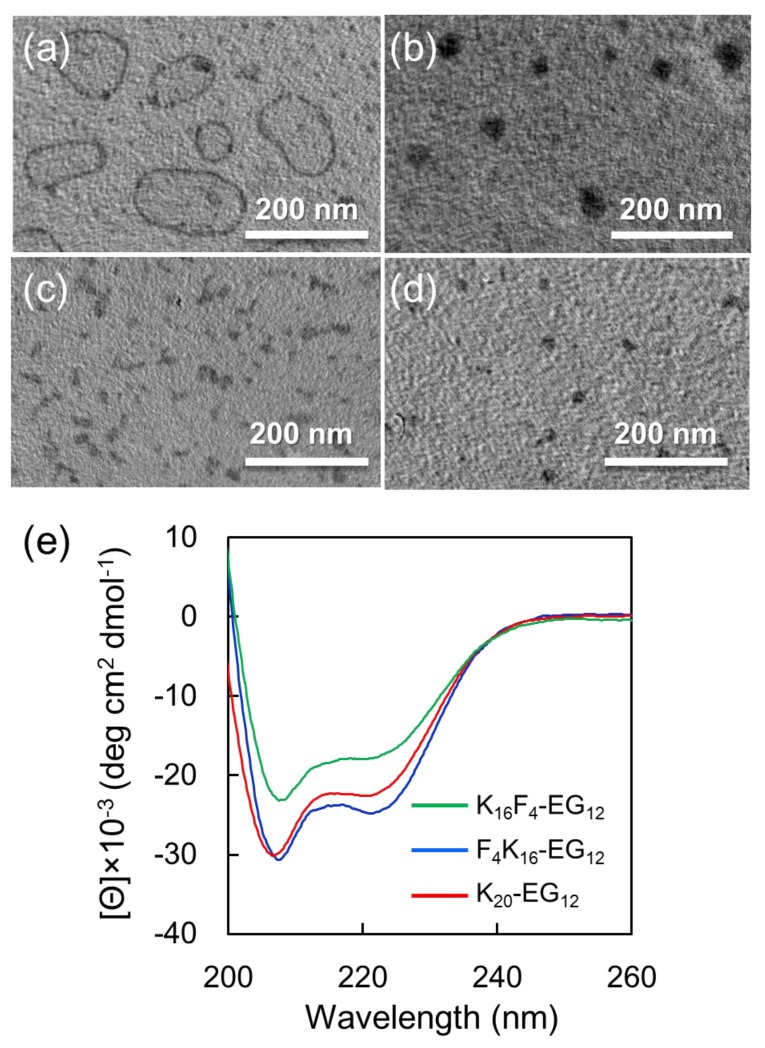
(**a**,**b**) Transmission electron microscopic (TEM) images of the nanostructures of F_4_K_16_-EG_12_ obtained in 25% TFE aqueous solution at pH 9.6 (**a**) and at pH 8.9 (**b**). (**c**) TEM image of the nanostructures of K_20_-EG_12_ obtained in 25% TFE aqueous solution at pH 9.3. (**d**) TEM image of the nanostructures of K_16_F_4_-EG_12_ obtained in 25% TFE aqueous solution at pH 9.7. (**e**) CD spectra of the nanostructures corresponding to (**a**) (blue), (**c**) (red), and (**d**) (green).

Next, we investigate the self-assembly of F_4_K_16_-EG_12_ in 25% TFE. A basic buffer solution was added dropwise to the TFE solution containing F_4_K_16_-EG_12_ peptide amphiphiles at the rate of 10 µL/min under gentle agitation until the concentration of TFE was 25%. The final concentration of the peptide was 3 mg/mL and the final pH value was 9.6. Under basic conditions, the peptide segments of F_4_K_16_-EG_12_ are hydrophobic, because the side amino groups of Lys residues are deprotonated. Thus, the addition of basic water would facilitate the self-assembly of F_4_K_16_-EG_12_ due to their amphiphilic molecular structures composing hydrophobic peptides and hydrophilic oligo (ethylene glycol)). The morphologies of obtained nanostructures were observed by TEM via drop deposition of the sample on Cu grid and staining with phosphotungstic acid. To prevent the structural change of the nanostructures during sample preparation for TEM observation, the staining reagent was dropped on the grid and then blotted immediately with filter paper. TEM images show that F_4_K_16_-EG_12_ formed nanostructures where a contrast between the dark periphery and the lighter center of spherical structures was observed, indicating that F_4_K_16_-EG_12_ formed vesicle like structures (Figure 2a, Figure S3a). The DLS measurement of the dispersion shows a monodisperse peak with an average diameter of 268.8 ± 72.2 nm (Figure S4a). The DLS date clearly indicates that the nanostructures were indeed formed in solutions. AFM observation revealed that the height of the nanostructures was much lower than the diameter of nanostructures that were estimated from DLS, indicating that nanostructures have hollow structures (Figure S5). In addition, it was confirmed that these nanostructures were stable at least for three days in 25% TFE solution.

The self-assembly of F_4_K_16_-EG_12_ in 25% TFE was further studied at various pH values. More importantly, vesicle formation successfully occurred at a narrow pH range (9.2–9.7) (Figure S6). At lower pH (8.9), large compound micelles with a diameter of 40 ± 8 nm were observed in preference to vesicles (Figure 2b, Figures S2b, S4b). The reason why vesicle formation is unlikely to occur in the lower pH range would be the low helical content of F_4_K_16_-EG_12_. This is consistent with the general consideration that side-by-side interactions of α-helical peptides between peptide-bond dipoles may induce vesicle formations [5,29].

The self-assembly of K_20_-EG_12_ or K_4_F_16_-EG_12_ was also evaluated in a similar manner as a control. The TEM image shows that K_20_-EG_12_ formed typical core-corona type micelles with a diameter of 10 ± 4 nm (Figure 2c, Figure S3c). Although both K_20_-EG_12_ and F_4_K_16_-EG_12_ adopt helical structures with helix content of over 90%, as estimated from CD spectra (Figure 2e), they provided nanostructures with different morphologies. These results suggest that hydrophobicity of tetra(phenylalanine) is significant to form vesicle structures. In addition, K_16_F_4_-EG_12_ forms core-corona type micelles with a diameter of 16 ± 5 nm, in addition to ill-regulated aggregates (Figure 2d, Figure S3d), indicating that the position of tetra(phenylalanine) is also significant for the formation of vesicle structures.

**Figure 3:**
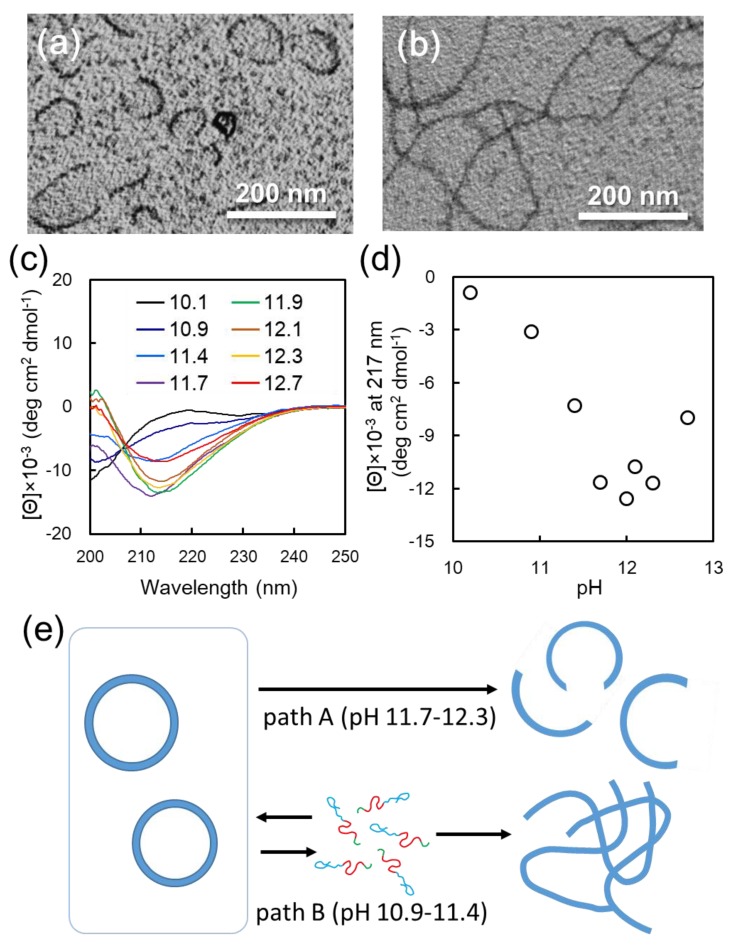
(**a**,**b**) TEM images of the nanostructures of F_4_K_16_-EG_12_ obtained by the dialysis of its vesicle dispersion against buffer solution at pH 12.0 (**a**) and at pH 10.9 (**b**). (**c**) CD spectra of F_4_K_16_-EG_12_ nanostructures obtained by solvent exchange at various pHs. (**d**) pH dependence of *θ*_217_ for the F_4_K_16_-EG_12_ nanostructures. (**e**) Schematic illustration of morphological transition of F_4_K_16_-EG_12_ from vesicles to arc-shaped nanostructures or long nanofibers.

It is expected that F_4_K_16_-EG_12_ vesicle structures will exhibit morphological transformation with the α-β transition of core-forming peptides when the solvent-environment was exchanged from 25% TFE aq. to water under basic condition, since there is a clear difference in molecular dimension between α-helix and β-sheet conformation. To change the solvent-environment of F_4_K_16_-EG_12_ vesicle, TFE was removed by the dialysis of F_4_K_16_-EG_12_ vesicle dispersion against basic buffer. Interestingly, when vesicle dispersion was dialyzed against carbonate buffer at pH 12, vesicle structures transformed to arc-shaped nanostructures (Figure 3a, Figure S7a). The arc-shaped nanostructures had relatively monodispersed size, in the range of 50–100 nm (Figure S8). AFM observation revealed that the height of arc-shaped nanostructures was about 5–8 nm (Figure S9). The transformation of the vesicle structures with solvent exchange was significantly affected by the pH of the buffer used for dialysis. The vesicle-to-arc transformation occurred by dialysis in the pH range of 11.7–12.3 (path A) (Figure S10). On the other hand, when dialysis was performed in a more acidic pH, ranging from 10.9 to 11.4 (path B), long nanofibers were formed (Figure 3b, Figure S7b). Dialysis below pH 10.9 or above pH 12.3 gave irregular aggregates (Figure S11).

To gain insight into the secondary structure of the nanostructures that were obtained after solvent-exchange by dialysis at various pH values from F_4_K_16_-EG_12_ vesicles, CD measurements were performed. The transition from α-helix to β-sheet conformation for all samples after dialysis was confirmed (Figure 3c). Based on these CD spectra, the peak intensity at 217 nm, representing to β-sheet conformation, was plotted as a function of pH in Figure 3d. This plot indicates that the β-sheet content notably increased in the pH range of 10.1–11.7, reached a maximum value, and decreased above pH 12.3. More importantly, the pH region of 10.9–11.4, where the β-sheet and random conformation are mixed, is consistent with that where long nanofibers were formed in morphological transition. On the other hand, the formation of arc-shaped nanostructures is observed in the pH region of 11.7–12.3, where the β-sheet content reaches saturation. 

Based on these results, we propose the mechanism behind vesicle-to-nanofiber and vesicle-to-arc transition, as follows (Figure 3e): Since the increase in β-sheet content of F_4_K_16_-EG_12_ with increasing pH is ascribed to the deprotonation of amino groups of Lys residues, it is reasonable to consider that the molecular mobility of F_4_K_16_-EG_12_ in water depends on its β-sheet content. Long nanofibers were formed by solvent exchanging in the pH region of 10.9–11.4 (path B). CD measurements revealed that the secondary structures of F_4_K_16_-EG_12_ were the mixture of random and β-sheet conformation in this pH region, indicating that amino groups of Lys residue would be assumed to be partially protonated. This moderate charge would allow the building block peptides to dissociate relatively easily from vesicle and to re-assemble into nanofibers accompanied by the transition into β-sheet conformation. This speculation is not inconsistent with the results that long nanofibers were formed by simply adjusting the pH of F_4_K_16_-EG_12_ aqueous solution to 10.9 (Figure S12a). On the other hand, the morphological change of the vesicle into an arc-shaped nanostructure was induced by dialysis in the pH region of 11.7–12.3 (path A), where the secondary structures of F_4_K_16_-EG_12_ are β-sheet rich conformation. In this pH region, the mobility of the building block peptides consisting of vesicles would be low, because amino groups of Lys residue would be deprotonated [30,31]. Therefore, through path B, α-β conformational transition of the building block peptides induced by solvent exchange would cause a shift in the type of hydrogen bonding—from intramolecular to intermolecular, i.e., between adjacent molecules. This would induce distortion in the vesicle, resulting in the collapse of vesicles and transformation to arc-shaped nanostructures. This postulated mechanism agrees with the results that pH adjustment of the F_4_K_16_-EG_12_ solution provided no arc-shaped nanostructures (Figure S12b).

## 4. Conclusions

We demonstrated the morphological transition of triblock PEG-peptide amphiphiles containing β-sheet forming an aromatic sequence from vesicles to arc-shaped nanostructures accompanied by α-β transition of core-forming peptides. The triblock PEG-peptide amphiphiles adopted an α-helix in 25% TFE aq., while β-sheet structures in water under basic pH condition. TEM observation revealed that the triblock amphiphiles formed vesicle structures in 25% TFE aq. When the solvent of vesicle dispersion was exchanged by dialysis against basic water, the vesicles were transformed into arc-shaped nanostructures with α-β conformational transition. We believe that the transformable nanostructures consisting of a building block with definite conformation both before and after transformation may be useful as a novel smart nanomaterial. Further characterization and functionalization of these vesicles consisting of triblock PEG-peptide amphiphiles is currently in progress.

## Figures and Tables

**Table 1:** Amino acid sequences of the PEG-peptide amphiphiles.

Abbreviation	Amino Acid Sequence
K_20_-EG_12_	KKKKKKKKKKKKKKKKKKKK-oligo(ethylene glycol)
K_16_F_4_-EG_12_	KKKKKKKKKKKKKKKKFFFF-oligo(ethylene glycol)
F_4_K_16_-EG_12_	FFFFKKKKKKKKKKKKKKKK-oligo(ethylene glycol)

## Supplementary Materials

The following are available online at http://www.mdpi.com/2073-4360/11/1/39/s1.
