# Supplementary material for: Morphological Transformation of Peptide Nanoassemblies through Conformational Transition of Core-forming Peptides
Source: Polymers (Basel). 2018 Dec 28;11(1):39. doi: 10.3390/polym11010039 (PMC6401806; doi:10.3390/polym11010039)
Supplement: Supplementary file 1 [file polymers-11-00039-s001.docx]

*Supporting Information*

Morphological Transformation of Peptide Nanoassemblies through Conformational Transition of Core-forming Peptides

*Tomonori Waku ^1,*^, Naoyuki Hirata ^1^, Masamichi Nozaki ^1^, Kanta Nogami ^1^, Shigeru Kunugi ^1^, and Naoki Tanaka ^1^*

^1^ Faculty of Molecular Chemistry and Engineering, Kyoto Institute of Technology, Gosyokaido-cho, Matsugasaki, Sakyo-ku, Kyoto 606-8585, Japan

*To whom correspondence should be addressed.

Faculty of Molecular Chemistry and Engineering, Kyoto Institute of Technology, Gosyokaido-cho, Matsugasaki, Sakyo-ku, Kyoto 606-8585, Japan. Tel.: +81 75 724 7811; Fax: +81 75 724 7861; E-mail: [waku1214@kit.ac.jp](mailto:waku1214@kit.ac.jp)

**Contents**

**Figure S1** (a) Helical content of K_20_-EG_12_ (circle), K_16_F_4_-EG_12_ (triangle), and F_4_K_16_-EG_12_ (square) in 25% TFE aqueous solution at various pHs. (b) Helical content of K_20_-EG_12_ in water at various pH values.

**Figure S2** pH dependence of *θ*_217_ for F_4_K_16_-EG_12_ (square) and K_16_F_4_-EG_12_ (triangle) in water.

**Figure S3** (a, b) TEM images of the nanostructures of F_4_K_16_-EG_12_ obtained in 25% TFE aqueous solution at pH 9.6 (a) and at pH 8.9 (b). (c) TEM image of the nanostructures of K_20_-EG_12_ obtained in 25% TFE aqueous solution at pH 9.3. (d) TEM image of the nanostructures of K_16_F_4_-EG_12_ obtained in 25% TFE aqueous solution at pH 9.7. Scale bars represent 200 nm.

**Figure S4** DLS histograms for the nanostructures of F_4_K_16_-EG_12_ in 25% TFE aqueous solution.: (a) vesicles, (b) large compound micelles.

**Figure S5** AFM images of nanostructures of F_4_K_16_-EG_12_ obtained in 25% TFE aqueous solution under basic condition. Cross-sectional images of the nanostructures.

**Figure S6** TEM images of the nanostructures of F_4_K_16_-EG_12_ obtained in 25% TFE aqueous solution at pH 9.2 (a, b) and at pH 9.5 (c, d). Scale bars represent 200 nm.

**Figure S7** TEM images of the nanostructures of F_4_K_16_-EG_12_ obtained by the dialysis of its vesicle dispersion against buffer solution at pH 12.0 (a) and at pH 10.9. Scale bars represent 200 nm.

**Figure S8** Size distribution of arc-shaped nanostructures consisting of F_4_K_16_-EG_12_. The number of nanostructures measured is 105.

**Figure S9** AFM image of arc-shaped nanostructures consisting of F_4_K_16_-EG_12_. Cross-sectional images of the nanostructures.

**Figure S10** TEM images of the nanostructures of F_4_K_16_-EG_12_ obtained by the dialysis of its vesicle dispersion against buffer solution at pH 11.9. Scale bar represents 200 nm.

**Figure S11** TEM images of the aggregates of F_4_K_16_-EG_12_ obtained by the dialysis of its vesicle dispersion against buffer solution at pH 10.1 (a) and at pH 12.7 (b).

**Figure S12** TEM images of the aggregates of F_4_K_16_-EG_12_ obtained by pH adjustment to pH 10.9 (a) and pH 12.1 (b) from pH 4 in water.


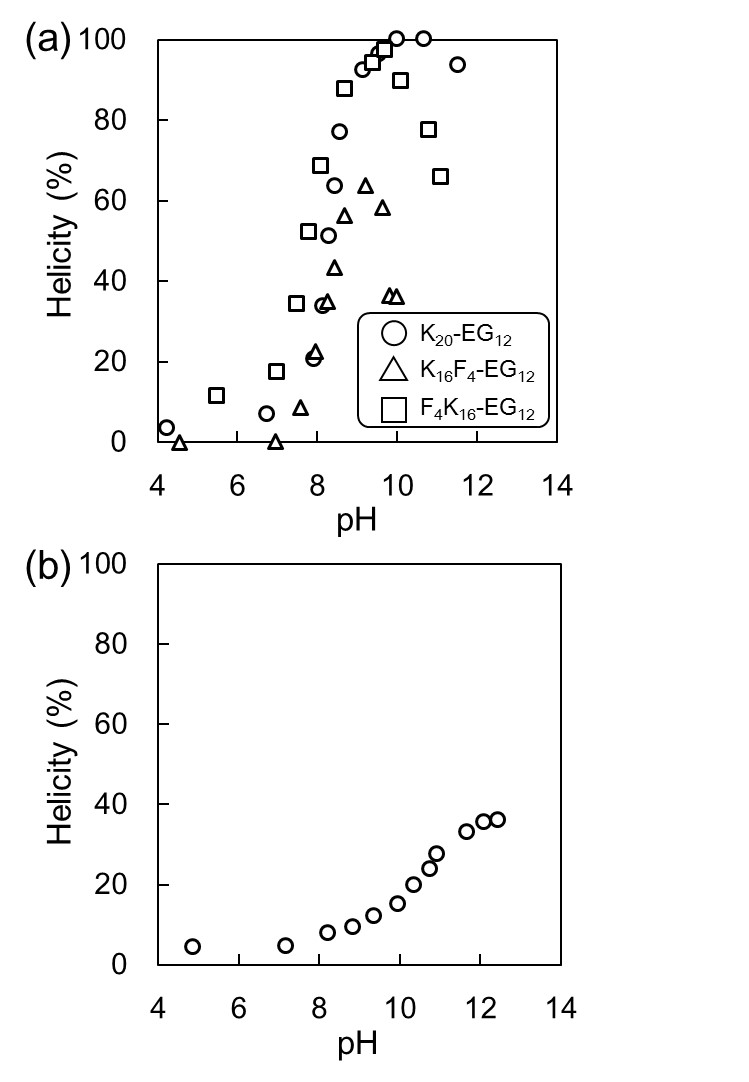


**Figure S1** (a) Helical content of K_20_-EG_12_ (circle), K_16_F_4_-EG_12_ (triangle), and F_4_K_16_-EG_12_ (square) in 25% TFE aqueous solution at various pHs. (b) Helical content of K_20_-EG_12_ in water at various pH values.


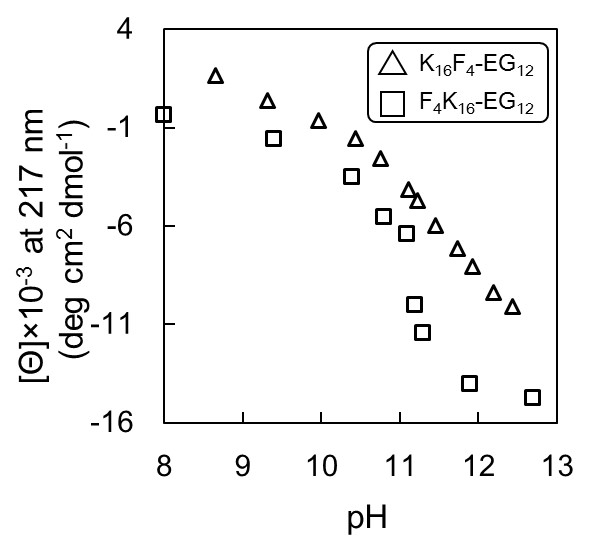


**Figure S2** pH dependence of *θ*_217_ for F_4_K_16_-EG_12_ (square) and K_16_F_4_-EG_12_ (triangle) in water.


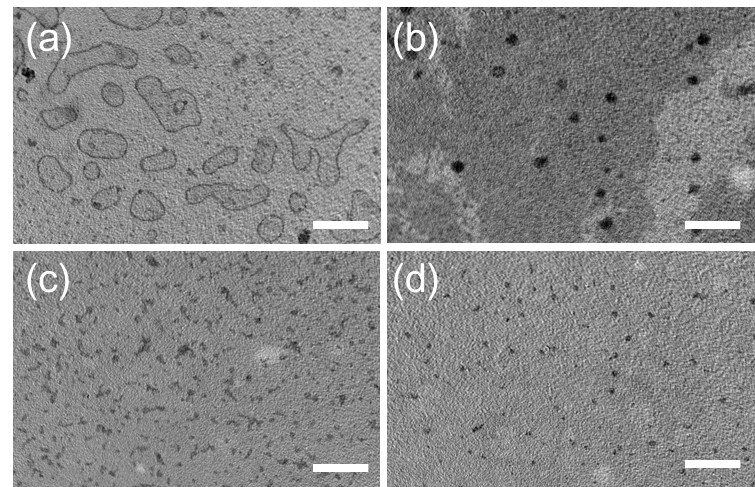


**Figure S3** (a, b) TEM images of the nanostructures of F_4_K_16_-EG_12_ obtained in 25% TFE aqueous solution at pH 9.6 (a) and at pH 8.9 (b). (c) TEM image of the nanostructures of K_20_-EG_12_ obtained in 25% TFE aqueous solution at pH 9.3. (d) TEM image of the nanostructures of K_16_F_4_-EG_12_ obtained in 25% TFE aqueous solution at pH 9.7. Scale bars represent 200 nm.


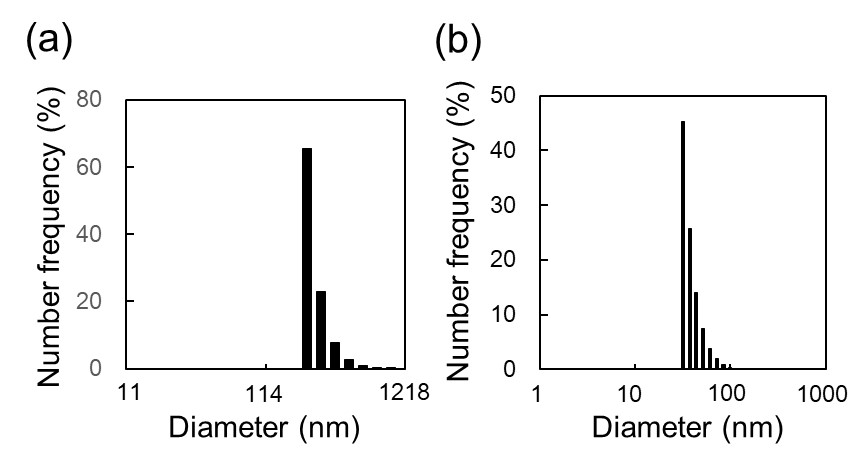


**Figure S4** DLS histograms for the nanostructures of F_4_K_16_-EG_12_ in 25% TFE aqueous solution.: (a) vesicles, (b) large compound micelles.


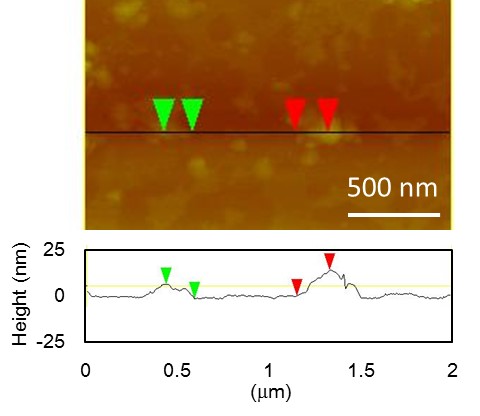


**Figure S5** AFM images of nanostructures of F_4_K_16_-EG_12_ obtained in 25% TFE aqueous solution under basic condition. Cross-sectional images of the nanostructures.


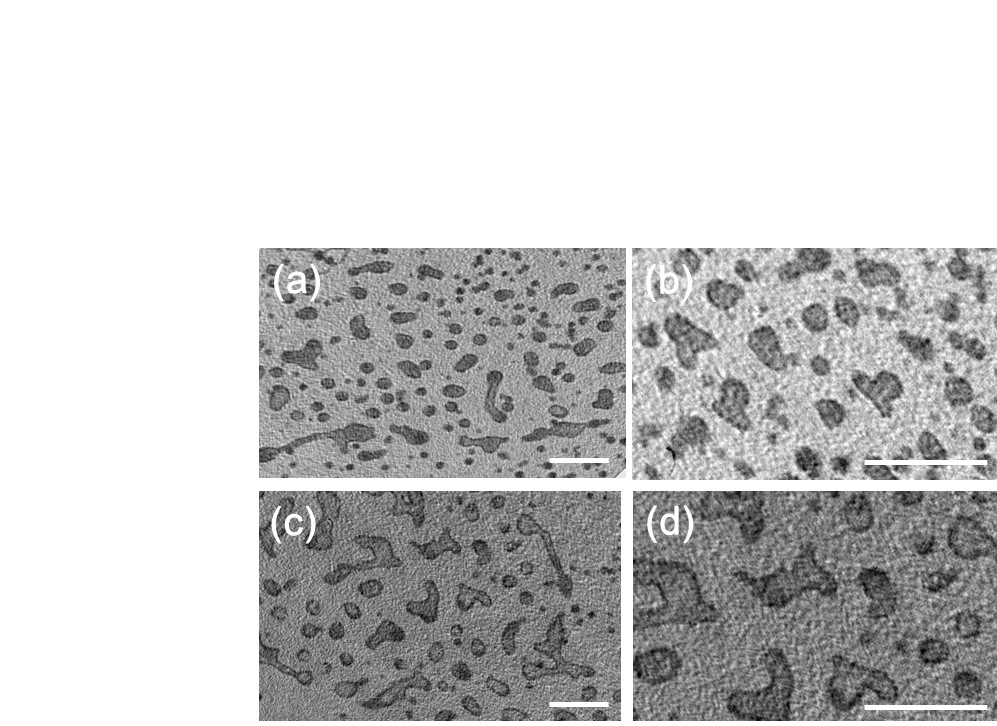


**Figure S6** TEM images of the nanostructures of F_4_K_16_-EG_12_ obtained in 25% TFE aqueous solution at pH 9.2 (a, b) and at pH 9.5 (c, d). Scale bars represent 200 nm.


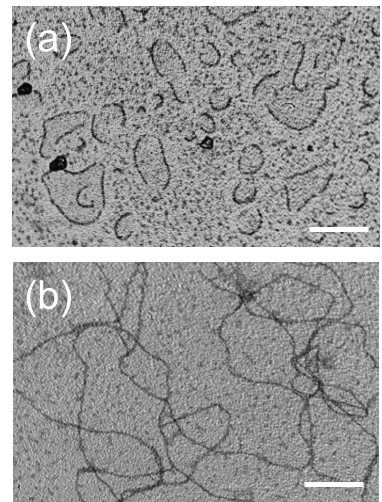


**Figure S7** TEM images of the nanostructures of F_4_K_16_-EG_12_ obtained by the dialysis of its vesicle dispersion against buffer solution at pH 12.0 (a) and at pH 10.9. Scale bars represent 200 nm.


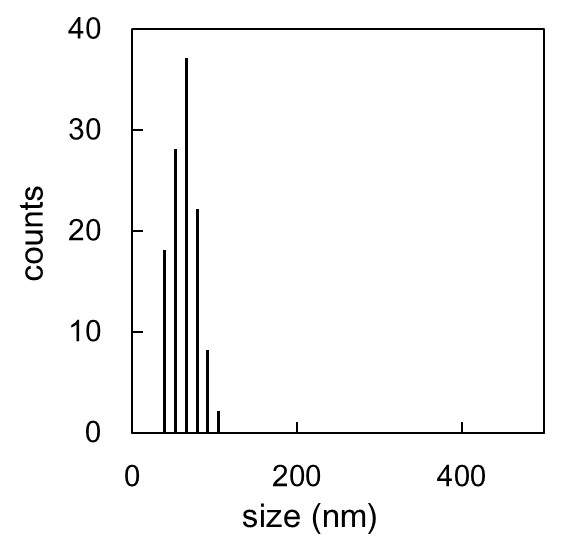


**Figure S8** Size distribution of arc-shaped nanostructures consisting of F_4_K_16_-EG_12_. The number of nanostructures measured is 105.

**Figure S9** AFM image of arc-shaped nanostructures consisting of F_4_K_16_-EG_12_. Cross-sectional images of the nanostructures.


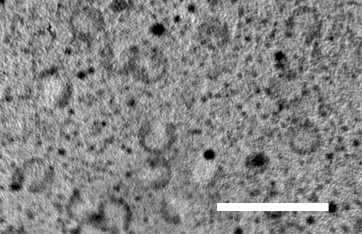


**Figure S10** TEM images of the nanostructures of F_4_K_16_-EG_12_ obtained by the dialysis of its vesicle dispersion against buffer solution at pH 11.9. Scale bar represents 200 nm.


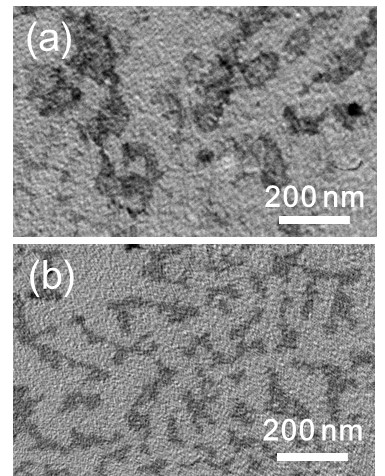


**Figure S11** TEM images of the aggregates of F_4_K_16_-EG_12_ obtained by the dialysis of its vesicle dispersion against buffer solution at pH 10.1 (a) and at pH 12.7 (b).


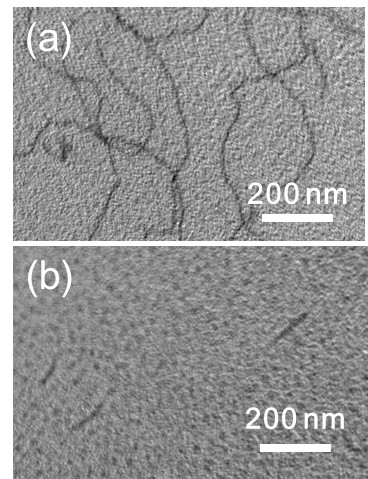


**Figure S12** TEM images of the aggregates of F_4_K_16_-EG_12_ obtained by pH adjustment to pH 10.9 a) and pH 12.1 (b) from pH 4 in water.
